# Anti-Adipogenic and Anti-Inflammatory Activities of (−)-*epi*-Osmundalactone and Angiopteroside from *Angiopteris helferiana* C.Presl

**DOI:** 10.3390/molecules25061337

**Published:** 2020-03-15

**Authors:** Ramakanta Lamichhane, Prakash Raj Pandeya, Kyung-Hee Lee, Se-Gun Kim, Hari Prasad Devkota, Hyun-Ju Jung

**Affiliations:** 1 Wonkwang University, College of Pharmacy, Dept. of Oriental Pharmacy, & Wonkwang-Oriental Medicines Research Institute, Iksan, Jeonbuk 54538, Korea; clickrama@hotmail.com (R.L.); pandeya.praj@gmail.com (P.R.P.); fuhaha112@naver.com (K.-H.L.); 2 Department of Agricultural Biology, National Academy of Agricultural Science, Rural Development Administration, Wanju 566-851, Korea; kimsegun@korea.kr; 3 Graduate School of Pharmaceutical Sciences, Kumamoto University, 5-1 Oe-honmachi, Kumamoto 862-0973, Japan; devkotah@kumamoto-u.ac.jp

**Keywords:** *Angiopteris helferiana*, (−)-*epi*-Osmundalactone, Angiopteroside, obesity, inflammation, adipogenesis, anti-inflammatory

## Abstract

*Angiopteris helferiana* C.Presl is a gigantic fleshy-type fern, belonging to Marattiaceae family. In previous study, we reported the potent anti-adipogenic and anti-inflammatory activities of ethylacetate (EtOAc) and *n*-butanol (BuOH) fractions of methanol extract of rhizomes of *A. helferiana*. In continuation, in this study, we report the isolation, characterization, and bioactivity analysis of principle bioactive compounds in these fractions. (−)-*epi*-Osmundalactone (**1**) and angiopteroside (**2**) were isolated from EtOAc and BuOH fractions, respectively. The structures of these compounds were established on the basis of NMR spectroscopic data. The quantification study using UPLC revealed the contents of compounds **1** and **2** in the dried rhizome to be 1.54% and 3.2%, respectively. These compounds were evaluated for their anti-adipogenic and anti-inflammatory activities using 3T3-L1 and RAW 264.7 cells, respectively. Compound **1** (2.5 µg/mL) and **2** (20 µg/mL) inhibited the lipid production by 35% and 25%, respectively. Regarding the anti-inflammatory activity, compound **1** (5 µg/mL) inhibited the nitrite production by nearly 82%. In conclusion, the presence of potent anti-adipogenic and anti-inflammatory compounds in *A. helferiana* indicate its potential role in the use of herb-based treatment for obesity and other related diseases.

## 1. Introduction

With the increasing modernization and lifestyle changes, metabolic disorder and related diseases are daily increasing and affecting a very large proportion of the global population. Obesity is one specific example and rapidly growing public health problem [1]. It has posed a great obstacle to social life, economies, and health systems of both developed and under-developed countries [2]. The world health organization (WHO) has announced that obesity has reached the epidemic proportion worldwide [3].

Obesity is mainly associated with the increase in number and size of adipogenic cells called “adipocytes” which store large amount of fat and lipids [4]. It is an important risk factor for diabetes, hypertension, and cardiovascular diseases along with some chronic diseases such as stroke, osteoarthritis, sleep apnea, some cancers, and inflammation-bases pathologies [5,6,7]. Obesity has also been linked with chronic low-grade inflammation which in the long run, leads to different chronic degenerative disorders. It induces the low-grade activation of the innate immune system which ultimately disturbs the steady-state measures of metabolic homeostasis over time. Excessive fat accumulation generally induce adipogenic macrophages to secrete different inflammatory cytokines (TNF-α and IL-6) which finally end up with obesity-induced inflammation [8,9]. The level of TNF-*α* increases with the size of adipose tissue. Higher level of TNF-*α* due to the increased level of free fatty acid reduces the insulin sensitivity, and, in the liver, it has an inhibitory effect on insulin action causing the increase in hepatic glucose production [10]. Leptin one of the major hormones secreted by adipocytes has important role in regulation of body weight through its effect on central nervous system for controlling the appetite. The majority of obesity patients show high level of leptin according to the degree of adiposity and hyperinsulinemia. This condition is referred to as leptin resistance [11]. Increased level of leptin or hyperleptinemia is mainly responsible for the insulin resistance which is very common in obese condition due to deterioration of function of insulin receptors in various cells [12]. Adiponectin is another major hormone secreted by the adipocytes that mainly increases the sensitivity of insulin apart from controlling the food intake. However, several studies have found that obese and diabetic patients show hypoadiponectinemia [13,14]. Adiponectin has various protective roles in the body like antidiabetogenic and antiatherogenic effect, along with its role in reducing the risk of increasing blood pressure, total cholesterol, and low density lipoproteins (LDL) [15,16]. Cross-sectional population studies have revealed that low adiponectin concentrations or high leptin levels are associated with higher chance of metabolic and cardiovascular problems [17,18].

The major treatment or preventive approach for obesity would be delaying the growth and expansion of adipocytes. Plant and plant-based phytochemicals have also been commonly used for the treatment of obesity apart from other allopathic medications. Several phytochemicals and active ingredients from plants are blessed with strong anti-obesogenic and anti-inflammatory properties. They play a special role in controlling weight gain by virtue of their antioxidant and anti-inflammatory properties [19]. Different mechanisms for the anti-obesity activity of the plant and plant-derived phytochemicals (flavonoids, polyphenols, terpenoids, alkaloids, glycosides) have been identified. They include: reduction in lipid absorption, decreased intake of energy, more expenditure of energy, reduced lipogenesis, inhibition of the lipase, and suppression of the adipose cell proliferation [20]. The antioxidant activity of phytochemicals has also been found to play an important role in the reduction of obesity-induced excessive accumulation of inflammatory cytokines [21]. So the plant and plant-derived phytochemicals have been highly effective in treating obesity and its complications [22].

**Figure 1:**
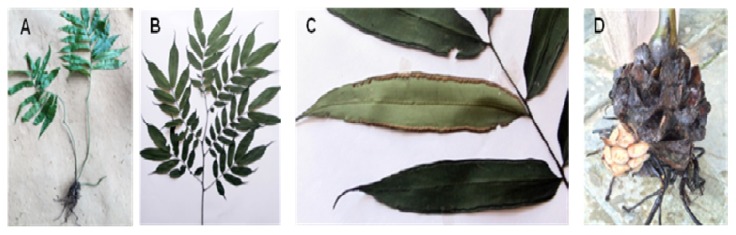
Photographs of different plant parts of *A. helferiana*: (**A**) Whole plant at early stage; (**B**) leaf (frond) of matured plant; (**C**) leaf bearing sori at the margin; and (**D**) rhizome.

*Angiopteris helferiana* C.Presl, a member of Marattiaceae family, is a gigantic fleshy fern with massive terrestrial bases with long and branch-like smooth stipes consisting of huge bipinnate leaves with long, narrow oblong pinnae bearing ultimate segments along their length and apically (Figure 1). The leaf consists of inframarginal sori on the ventral surface. *A. helferiana* is distributed in moist forest and slopes in an altitude of 900–1400 m in Nepal, China, India, Sri Lanka, and S.E. Asia [23,24]. In the Konkan region of Maharashtra, India, the rhizome of this plant is reported to be used for the treatment of scabies [25]. Various other species of the genus *Angiopteris* are used for medicinal purposes for the treatment of snake bite, rheumatism, cough, and pain [26]. For example, *A. esculenta* Ching (endemic to north-west of Yunnan Province, China) was reported to be used as a foodstuff by the local people for the extraction of starch. From the extracts of the aerial parts of the *Angiopteris* plants different lactones and coumarin have been isolated and identified [26]. In previous study, we reported that the ethylacetate (EtOAc) and *n*-butanol (BuOH) fractions of methanol extract of rhizomes of *A. helferiana* showed potent anti-adipogenic and anti-inflammatory activities [27]. As a continuation, in this study we report the isolation, characterization, and bioactivity analysis of principle bioactive compounds in these fractions.

## 2. Results and Discussion

### 2.1. Isolation and Identification of Compounds

The dried rhizomes of *A. helferiana* were extracted with MeOH by using a reflux condenser. The extract was filtered and dried under reduced pressure using rotary evaporator to obtain MeOH extract. The extract was then suspended in 1000 mL of water and partitioned successively with hexane, dichloromethane, EtOAc, and n-BuOH to obtain respective fractions i.e., hexane-, dichloromethane-, ethyl acetate-, *n*-butanol-, and water fractions [27]. The ethyl acetate fraction was further purified using reverse phase column chromatography, normal phase chromatography, and Sephadex LH-20 column chromatography to obtain compound **1**. Compound **2** was isolated from the *n*-BuOH fraction after subjecting to repeated column chromatography on Sephadex LH-20.

**Figure 2:**
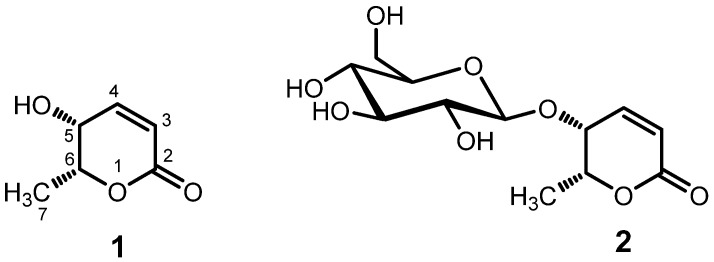
Structures of (−)-*epi*-osmundalactone (**1**) and angiopteroside (**2**).

Compound **1** was obtained as a colorless liquid, [α]_D_^22^ −109.4 (c 1.28, H_2_O). The UV spectrum of compound **1** showed absorption maxima (λ_max_) at 210 nm. The ^1^H-NMR spectrum of compound **1** in in DMSO-*d*_6_ (Table 1) showed proton signals for two olefinic protons at δ_H_ 7.01 p (1H, dd, *J* = 9.6, 5.7 Hz) and 5.98 ppm (1H, d, *J* = 9.6 Hz), two methine protons at δ_H_ 4.48 (1H, qd, *J* = 6.6, 2.8 Hz) and δ 3.93 ppm (1H, m), and a methyl group at δ_H_ 1.29 (3H, d, *J* = 6.6 Hz), which were similar to that of 5,6-dihydro-5-hydroxy-6-methyl-2H-pyran-2-one [26]. The small coupling constant of 2.8 Hz between the protons attached to C-5 and C-6 suggested the cis-orientation of these proton as the coupling constant for these protons in trans-orientation is reported to be about 6 Hz [26,28,29] Comparing the obtained data with the published articles, the structure of compound **1** was elucidated as (−)-*epi*-osmundalactone [(5*R*,6*R*)-5,6-dihydro-5-hydroxy-6-methyl-2H-pyran-2-one] as shown in Figure 2 [26]. The ^13^C NMR spectrum of compound **1** displayed total of six carbon signals that were also superimposable with the reported values [26,28]. The *epi*-osmundalactone moiety was reported from nature in the form of its glucoside, angiopteroside from *Angiopteris lygodiifolia* Ros. [30] and also as a dual-lactone compound, angiopterolactone A from *Angiopteris caudatiformis* Hieron [31]. Recently, its isolation in pure form from natural source (*Angiopteris esculenta* Ching) was reported by Chen et al. in 2010 [26]. Similarly, the stereoselective synthesis of **1** has also been reported by Blume et al. [28]. On the other hand, (−)-osmundalactone having 5R,6S-configuration was previously isolated and identified from the fern *Osmunda japonica* Thunb [29] and (+)-osmundalactone having 5S,6R-configuration was reported from a fungus, *Paxillus atromentosus* [32].

Compound **2** was isolated as a white amorphous powder, [α]_D_^22^ −240.0 (c 0.46, H_2_O). The ^1^H- and ^13^C-NMR data of compound **2** were similar to compound **1**, except for the appearance of NMR signals of glucosyl moiety (Table 1). The anomeric proton at δ_H_ 4.3 ppm with coupling constant (*J*) of 7.8 Hz indicated the presence of a β-glucoside linkage in the structure. The ^13^C-NMR spectra of compound **2** showed signals equivalent to total 12 carbons, in which six carbon signals were assignable to a substituted (−)-*epi*-osmundalactone (**1**) and other six signals were assignable to glucopyranosyl moiety. It was further confirmed by performing the hydrolysis of the compound **2** in 0.1% sulfuric acid and ethanol (1:1) and co-TLC of reaction mixture with standard glucose using the solvent system of *n*-butanol, acetic acid, and water (4:1:5, organic phase). By comparing these data with literature, the structure of compound **2** was elucidated as angiopteroside [31]. Angiopteroside had been previously isolated and identified from the ferns, *Angiopteris lygodiifolia* Ros. [31], *Angiopteris evecta* (G. Forst.) Hoffm. [33,34,35], and *Angiopteris esculenta* Ching [26]. Its diasteromer osmundalin, a glucoside of (−)-osmundalactone, was reported from a fern, *Osmunda japonica*, and it showed stimulating effect on the deterrent cell of *Bombyx mori* [29,36].

### 2.2. Quantification of Isolated Compounds in Plant A. helferiana

The quantification of compounds in a plant extract would help any researchers to evaluate how abundant are those compounds in that plant. The UPLC analysis was used for the quantification of the compounds **1** and **2** in the methanol extract of *A. helferiana*. The retention time and the UV spectra of standard compounds were evaluated for the identification of peaks in the chromatogram of methanol extract of *A. helferiana*.

**Figure 3:**
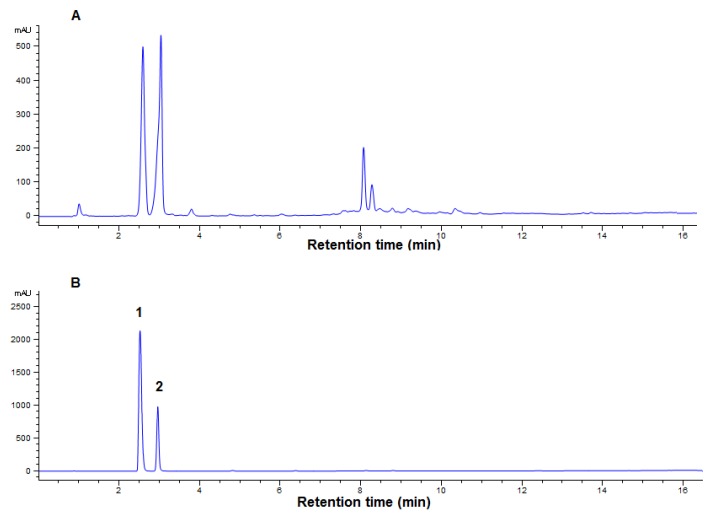
Ultra performance liquid chromatography (UPLC) chromatograms of methanol extract of *A. helferiana* and standard-mixture (compounds **1** and **2**). A suitable UPLC condition was established and the UPLC chromatogram of methanol extract (5 mg/mL) of *A. helferiana* (**A**) was obtained. The standard compound **1** and **2** were mixed (1 mg/mL each) and the UPLC chromatogram was obtained under similar condition (**B**) maintaining same column condition and solvent system. The peaks in chromatogram of methanol extract were identified as compounds **1** and **2** peaks after comparing with the retention time and UV-spectrum with that of standard compound peaks in chromatogram B.

A chromatogram of methanol extract of *A. helferiana* (Figure 3A) with better resolution of peaks was obtained using the suitable UPLC condition as described in the material and method section. Under similar UPLC condition, individual compounds **1** and **2** (1 mg/mL each) were injected separately and the retention times of compound **1** and **2** were found to be 2.6 and 3.0 min, respectively (Figure not shown). Then, the mixture of compounds **1** and **2** was injected and analyzed under similar UPLC conditions and their chromatogram is represented in Figure 3B with the retention times of 2.6 and 3.0 min, respectively. Thus, the peaks in the chromatogram of methanol extract of *A. helferiana* (Figure 3A) at 2.6 and 3.0 min were identified as compound **1** and **2** after comparing their retention time and UV-spectra with the peaks of standard-mixture. Standard curves for each compound **1** and **2** were prepared and their regression equation was obtained (Table 2). The quantification of compound **1** and **2** in the extract was done from the regression equation of the calibration curves and the peak areas of the respective compound in the extract. The results showed that the dried rhizome of *A. helferiana* contained around 1.54% and 3.2% of compound **1** and **2,** respectively (Table 2).

### 2.3. Anti-Adipogenic Activity of Isolated Compounds

**Figure 4:**
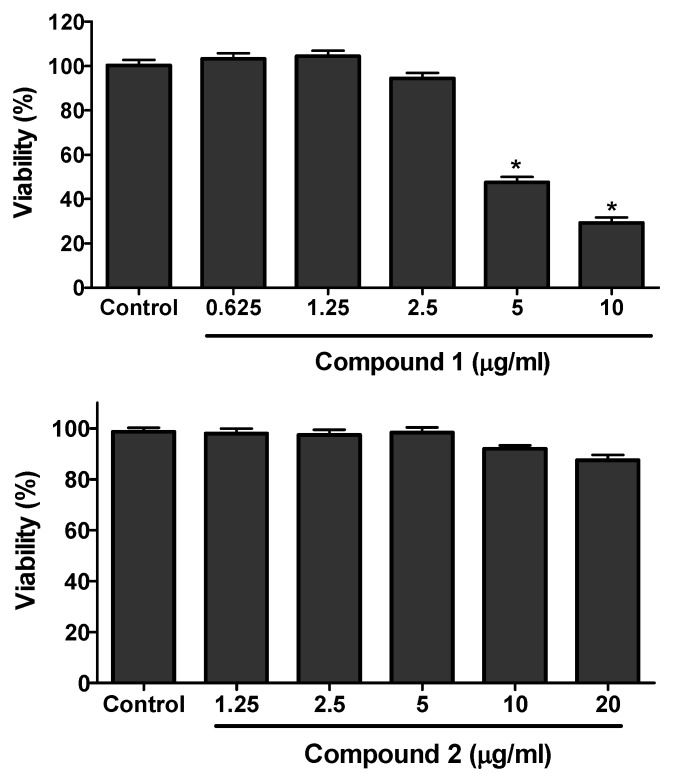
3T3-L1 cell viability assay of compounds **1** and **2**. The preadipocytes cells of 3T3-L1 were cultured and seeded in 96-well plate. After two days of confluence, the cells were treated with or without different concentration of compounds **1** and **2** and cell viability was determined by MTT-assay. The data show relative mean viability percentage ± SD of triplicate experiments. Statistical significance was calculated using one-way ANOVA followed by Dunnett’s test. * *P* < 0.05 vs. control.

The cell viability assay was performed for the isolated compounds against 3T3-L1 cells using the MTT assay. Concentrations ranging from 0.625 to 40 µg/mL were used for the viability study. Compound **1** was found toxic above 2.50 µg/mL whereas compound **2** was toxic above 20 µg/mL (Figure 4).

**Figure 5:**
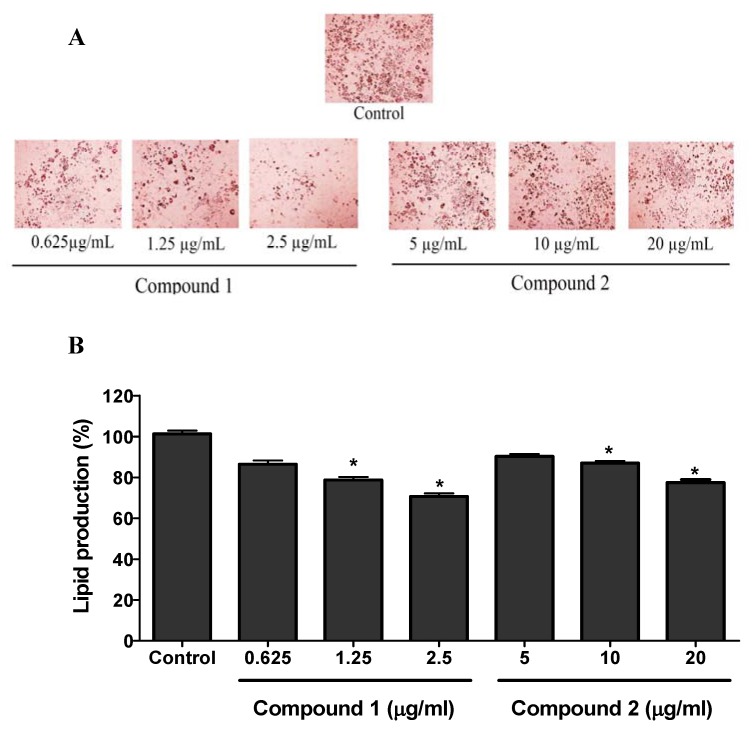
Anti-adipogenic activity of compounds **1** and **2**. 3T3-L1 preadipocytes were cultured and differentiated using differentiating media. Cells were differentiated with or without the presence of compounds **1** and **2**. The production of lipid in the cells was observed by using oil red O (ORO) staining agent (**A**). The ORO stain (red pigments) in the cells were eluted in isopropanol and evaluated for the quantification of lipid production in the cells from each group (**B**). The data show relative mean lipid production percentage ± SD of triplicate experiments. Statistical significance was calculated using one-way ANOVA followed by Dunnett’s test. * *P* < 0.05 vs. control.

The non-toxic concentrations of compound **1** (0.625, 1.25, and 2.5 µg/mL) and compound **2** (5, 10, and 20 µg/mL) to the 3T3-L1 cells were used for the anti-adipogenic activity study. 3T3-L1 cells were cultured in 6-well plates and differentiated using differentiation media with or without the presence of compounds **1** and **2**. The lipid produced in the cells was stained with ORO (oil-red-O) solution as depicted in the Figure 5A by the red pigments. Both compound **1** and **2** showed the significant inhibition of lipid production in 3T3-L1 cells. The results in Figure 5B showed the quantitative data for the lipid production in the sample (compounds **1** and **2**) treated and control groups. Compared to the control, compound **1** showed maximum lipid inhibition of around 30% (at 2.5 µg/mL) while compound **2** showed around 20% lipid inhibition at 20 µg/mL.

**Figure 6:**
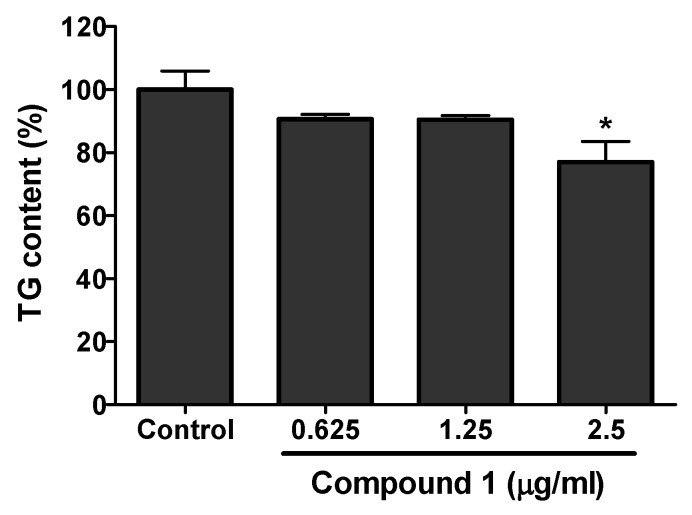
Total triacylglycerol (TG) content in the 3T3-L1 cells with or without the treatment of compound **1**. Cells were cultured and seeded in 6-well plated. Cells were differentiated using differentiation media in presence or absence of compound **1**. The cells were lysed and TG content was evaluated using the kit. The significant inhibition of TG content compared to the control was seen in the highest concentration used in the analysis. The data show relative mean TG content percentage ± SD of triplicate experiments. Statistical significance was calculated using one-way ANOVA followed by Dunnett’s test. * *P* < 0.05 vs. control.

Compound **1** having greater anti-adipogenic activity from the above experiment was further studied for its effect on triacylglycerol (TG) level in 3T3-L1 cells. The effect on TG content in the differentiated 3T3-L1 cells with or without the presence of isolated compound was analyzed. Compound **1** showed the significant inhibition of TG in the differentiated 3T3-L1 cells. A maximum of 20% inhibition of TG compared to the control was shown at a concentration of 2.5 µg/mL by compound **1** (Figure 6).

### 2.4. Anti-Inflammatory Activity of Isolated Compounds

**Figure 7:**
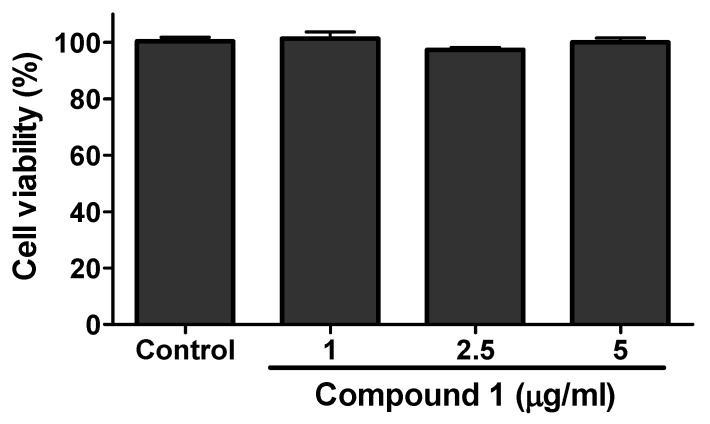
RAW cells viability assay of compound **1**. Raw cells were cultured and seeded in 96-well plate. After more than 80% confluence, the cells were treated with different concentration of compound **1** and cell viability was determined using MTT-assay. The data show relative mean expression ± SD of triplicate experiments.

**Figure 8:**
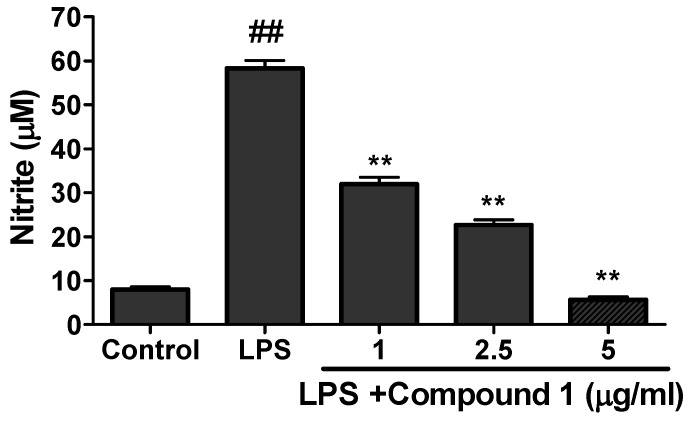
Production of nitrite ions after the treatment of compound **1**. RAW cells were cultured and seeded in 96-well plate. After that the cells were exposed to LPS and followed by treatment of different concentration of compound **1**. The production of nitrite ion in presence or absence of samples was evaluated using Griess reagent. The data show relative mean expression ± SD of triplicate experiments. Statistical significance was calculated using one-way ANOVA followed by Dunnett’s multiple comparisons test. ^##^ *P* < 0.01 vs. Control; ** *P* < 0.01 vs. LPS.

The viability assay of compounds **1** and **2** was evaluated against RAW 264.7 cells by MTT assay. The results of viability assay for compound **1** are given in Figure 7. Compound **1** was found to be toxic above the concentration of 5 µg/mL. Compound **2** was found toxic above 40 µg/mL (data not shown). For the evaluation of anti-inflammatory activity, the cultured RAW 264.7 cells were treated with LPS (endotoxin to induce inflammation in cells) in the presence or absence of different concentration of samples (Compounds **1** to **2**). Neither sample nor LPS was treated to the control. The production of nitrite was evaluated by Griess reagent and quantified from the standard curve of sodium nitrite. As shown in the Figure 8, the higher concentration of nitrite ion was seen in the LPS-only treated group compared to the control indicating the state of inflammation induced in the RAW cells. However the treatment of compound **1** showed significant inhibition of nitrite production in the LPS-treated group. Compound **1** inhibited the production of nitrite ion up to 80% compare to the LPS-only treated group (Figure 8). Compound **2** did not show any significant inhibition even at the higher dose of 40 µg/mL (results not shown).

The anti-adipogenic study confirmed that both compounds have lipid inhibiting activity in 3T3-L1 cells. (−)-*epi*-Osmundalactone showed higher lipid inhibiting potency than angiopteroside. The results of anti-inflammatory activity on RAW cells also showed a strong activity of (−)-*epi*-osmundalactone compared to angioptereoside. Analyzing the biological activity and structure of both compounds, it was observed that the presence of glucosidic linkage could diminish the biological activity as the angiopteroside having glucosidic linkage showed weaker biological activity compared to (−)-*epi*-osmundalactone. Different studies have also illustrated the difference in activity of certain compounds in glycoside and aglycone form. In a previous study, quercetin and quercetin glycosides were found to show different biological activities [37]. This study also supported the change in biological activities of natural flavonoid glycosides compared to their aglycone moiety. However, the two isolated compounds (−)-*epi*-osmundalactone and angiopteroside showed anti-adipogenic and anti-inflammatory activity and to the best of our knowledge this is first time being reported for those compounds. Further study of the compounds in animal model is required for the evaluation of in vivo anti-obesity activity. This would further strengthen the possibility of the compounds to be used as anti-obesity medications.

## 3. Conclusions

From the bioactive fractions of methanol extract of rhizomes of *A. helferiana*, two compounds (−)-*cis*-osmundalactone and its glucoside, angiopteroside were isolated for the first time. Their biological activities were also studied and we revealed that (-)-*cis*-osmundalactone (**1**) showed potent anti-adipogenic and anti-inflammatory activities whereas angiopteroside (**2**) showed good anti-adipogenic activity. The quantification study using UPLC indicated that *A. helferiana* is a good source for both of these compounds. Overall, *A. helferiana* also possessed potent biological activities and could be a major component for different herbal medications. However, further in vivo studies regarding the pharmacological effects and possible toxicity of these extracts and compounds are necessary to access the therapeutic potential.

## 4. Material and Methods

### 4.1. General Experimental Procedure

^1^H- and ^13^C- NMR spectra were measured in DMSO-*d*_6_ on BRUKER AVANCE 600 NMR Spectrometer (Bruker, Billerica, MA, USA) (^1^H-NMR: 600 Hz and ^13^C-NMR: 150 Hz). Chemical shift values (*δ*_H_ and *δ*_C_) are given in ppm with reference to tetramethylsilane (TMS). Column chromatography (CC) was carried out with Sephadex LH-20 (Amersham Pharmacia Biotech, Tokyo, Japan), ODS (ODS-A (12 nm, S-75 μm, YMC, Kyoto, Japan), and silica gel 60 (Kieselgel 60, 70–230 and 230–400 mesh, Merck, Darmstadt, Germany). Thin layer chromatography (TLC) was performed on a pre-coated silica gel 60 F_254_ (Aluminum sheet, Merck KGaA, Darmstadt, Germany). Optical rotations were measured with a JASCO DIP-1000 KUY polarimeter. The quantification of isolated compound was performed using ultra performance liquid chromatography (UPLC) system (Agilent Technologies 1290 Infinity) equipped with a quaternary pump (ACQ-QSM), auto-sampler, and photo diode array detector (ACQ-PDA).

### 4.2. Reagents and Cells

Solvents including ethanol, methanol, ethyl acetate, hexane, *n*-butanol, dichloromethane were purchased from SK Chemicals Co. Ltd. (Seongnam, Korea). Dimethyl sulfoxide (DMSO) was purchased from Junsei Chemicals Co. Ltd. (Tokyo, Japan), and sulfanilic acid, *N*-(1-napthyl) ethylenediamine dihydrochloride, was purchased from Sigma-Aldrich (MO, USA). Cell experiments: RAW 264.7 macrophage cells and 3T3-L1 preadipocytes cells were obtained from the American Type Culture Collection (Rockville, MD, USA). Dulbecco’s modified eagle medium (DMEM), newborn calf serum (NCS), and fetal bovine serum (FBS) were obtained from Gibco-Thermo-Fisher Scientific (Grand Island, NY, USA). 3-Isobutyl-1-methylxanthine (IBMX), dexamethasone, insulin, 10% formalin, isopropanol, and oil red O (ORO) were purchased from Sigma-Aldrich (St. Louis, MO, USA). Thiazolyl blue tetrazolium bromide (MTT), used to assess cell viability, was purchased from Alfa Aesar (Tewksbury, MA, USA).

### 4.3. Plant Material

The rhizomes of *Angiopteris helferiana* C. Presl. were collected from a forest at Jamune, Tanahun District, Nepal in August 2015 and identified by Mr. Dhan Raj Kandel, Assistant Research Officer, National Herbarium and Plant Laboratories, Godawari, Lalitpur, Nepal. A voucher specimen (Specimen No. 2) of the plant has been preserved in the Herbarium of National Herbarium and Plant Laboratories, Godawari, Lalitpur, Nepal. They were washed and shade dried for two weeks. The rhizomes were cut into small pieces before extraction.

### 4.4. Extraction, Fractionation, and Isolation of Compounds

The dried rhizomes (1.56 kg) were put in a flask with MeOH and were heated at 40–50 °C for 4–5 h using reflux condenser. The extract was filtered and dried under reduced pressure using rotary evaporator to obtain crude MeOH extract (310 g). The extract was then suspended in 1000 mL of water and partitioned successively with hexane, dichloromethane, ethyl acetate, and 1-butanol to obtain respective fractions, i.e., hexane- (3.1 g), dichloromethane- (8.0 g), ethyl acetate- (16.7 g), 1-butanol-(49.8 g), and water fractions (182.0 g) [27].

The ethyl acetate fraction was loaded on a reverse phase column chromatography. The column was eluted with methanol (5 to 100%). The eluting solvents were decided according to the separation required after monitoring by TLC. These elutes were collected and concentrated to a residue. The residue having similar TLC patterns were collected and dried to give eleven fractions (**a** to **k**). Fraction “**a**” was dried and loaded on a normal phase silica gel column and the column was eluted with solvent system of hexane and EA (2:8). Total 28 fractions (each 100 mL) were collected that were later mixed according to the similarity in the TLC pattern to give four major fractions (I to IV). Major fraction II (fractions 9–15) contained single spot that was again dried and subjected to Sephadex LH-20 column chromatography with gradient solvent system of water and methanol (water 100% to 0%) and compound **1** (80 mg) was isolated.

Similarly, the butanol fraction was subjected to Sephadex LH-20 column chromatography and eluted with gradient solvent system of methanol and water (methanol 10 to 80%). Altogether 45 fractions were collected (100 mL/fraction). On the basis of TLC patterns, these fractions were mixed to give seven major fractions: I to VII. Major fraction I was again subjected to Sephadex LH-20 column chromatography and compound **2** was isolated. Amorphous powder was again dissolved in methanol and left overnight for crystallization. After 24 h, crystals of compound **2** (60 mg) were formed that were washed with chloroform and dried using vacuum.

### 4.5. Quantification of Compounds **1** and **2** in Extract of rhizomes of A. helferiana

A suitable UPLC method was established for the better resolution of the peaks in the chromatogram of the methanol extract of *A. helferiana*. Total of 5 mg/mL methanol extract of *A. helferiana* prepared in methanol was injected in the UPLC system and a suitable solvent system was evaluated for the better resolution of the analyte peaks. The column, solvent system and other conditions regarding optimum UPLC condition are given as follow:

- Column—Halo C18, 2.7 μm, 4.6 × 100 mm
- Solvent system—Acetonitrile and 0.1% H3PO4 in Water
- Injection volume—2 μL
- Column temperature—40 °C
- Flow rate—1 mL/min
- Gradient Solvent system: Acetonitrile: 0% (0 min), 10% (6 min), 15% (6.5 min), 20% (10 min), 30% (12 min), 50% (14 min), 100% (16 min)
- UV absorption—220 nm

Total of 1 mg/mL of compounds **1** and **2** was injected separately under similar UPLC condition. Comparing the retention time and uv-spectrum of standard compounds, the peaks of methanol extract were identified. Stock solution of standards (compounds **1** and **2**) were diluted to appropriate concentration in order to plot calibration curves. The peak area of the analytes in the chromatogram of methanol extract of *A. helferiana* was used for the quantification.

### 4.6. Cell Viability Assay

Cell viability was determined colorimetrically using MTT assay. Raw 264.7 cells were cultured overnight in 96-well plate at a density of 5 × 10^4^ cells/200 μL in each well. 3T3-L1 pre-adipocytes were cultured in 48-well plate at a density of 2.5 × 10^4^ cells/well. Raw 264.7 cells and 3T3-L1 cells were cultured in DMEM with 10% FBS and 10% NCS respectively at 37 °C in 5% CO_2_. After 24 h, medium was changed with 10% NCS or 10% FBS DMEM and the cells were treated with the compound at a final concentration of 0.625 µg/mL to 40 µg/mL (in DMSO) for 2 days. The control received the same amount of DMSO. The final concentration of DMSO in the medium was not more than 1%. Then MTT solution was added to the each well with final concentration 1 mg/mL and incubated for 4 h. Formazan formed by viable cells was dissolved with DMSO and absorbance was determined at 520 nm by ELISA reader.

### 4.7. Anti-Adipogenic Activity (Differentiation of 3T3-L1 cells) Assay

3T3-L1 pre-adipocytes were cultured in DMEM with 10% bovine calf serum at 37 °C in a humidified atmosphere of 5% CO_2_. Differentiation media (MDI) i.e., DMEM medium containing 0.5 mM IBMX, 1 μM Dexamethasone, 5 μg/mL insulin, and 10% FBS, was put two days after the 100% confluence (Day 0), to initiate adipocyte differentiation. Two days after the induction of differentiation, on Day 2, the culture medium was changed with DMEM supplemented with only 5 μg/mL insulin and 10% FBS. After that the medium was changed on every 2 days (Day 4, Day 6, and Day 8) with DMEM containing only 10% FBS. The cells were treated with compound 1 to 5 at Day 0 and Day 2 in their non-toxic concentration as obtained from the viability study. At Day 10 Oil Red-O staining was done to determine the lipid production.

### 4.8. Oil Red O Staining Assay

Cells were washed twice with 1X PBS, fixed in 10% formaldehyde for 30 min, and then washed with 60% isopropanol and then stained with the oil red O working solution (6:4, 0.6% Oil Red O dye in isopropanol: water) for 30 min at room temperature and washed three times with tap water. Staining was visualized by bright-field microscopy, and oil red O dyes extracted from the cells in isopropanol were quantified at a wavelength of 520 nm.

### 4.9. Triacylglycerol (TG) Content Assay

The intracellular triglyceride was assessed on Day 8 of differentiation. After washing tow times with ice-cold PBS, the cells were collected using 0.1 mL of cell lysis buffer and scraping the cell with a cell scraper. The collected cells were then vortex for 5 min and centrifugation at 15,000 g for 15 min at 4 °C. The supernatants were assayed for triglyceride content according to the manufacturer’s protocol [TG-S (Triglyceride) kit] with some modification. Triglyceride assay buffer (180 µL) and cell lysate (50 µL) was mixed and incubated for 1 h at 37 °C and then absorbance was measured at 540 nm wavelength using a micro-plate reader.

### 4.10. Anti-inflammatory Activity Assay

Nitrite ions that accumulated in the cultural medium were measured as an indicator of NO production based on the Griess reaction. In brief, RAW 264.7 cells were plated in a 24 well plate at a density of 50 × 10^4^ cells/500 μL in each well. Then after 24 h of incubation in95% air and 5% CO_2_ humidified atmosphere at 37 °C the cells were treated with different concentration of compound **1** to 5. After 1 h, cells were treated with LPS (1 μg/mL) in both compound treated as well as untreated wells. Amounts of nitrite, a stable metabolite of NO, were measured using Griess reagent (1% sulfanilamide and 0.1% napthylethylenediamine dihhydorchloride in 2.5% phosphoric acid). Briefly, 100 μL of cell culture medium was mixed with 100 μL of Griess reagent. Subsequently, the mixture was incubated at room temperature for 10 min and the absorbance at 540 nm was measured in a microplate reader. Fresh culture media was used as a blank. The quantity of nitrite was determined from a sodium nitrite standard curve.

### 4.11. Statistical Analysis

Statistical analysis was done by using graphpad prism 6 software (GraphPad Software, San Diego, CA, USA). One-way analysis of variance (ANOVA) using Dunnett’s test was performed to compare the each of the test samples to the single control. For multiple comparisons between different groups one-way ANOVA was applied using Tukey’s multiple comparison test. *P* value < 0.05 was considered to be significant.

## Figures and Tables

**Table 1:** ^1^H and ^13^C NMR data of compound **1** and **2** in DMSO-*d*_6._

Position	(−)-*epi*-Osmundalactone (1)		Angiopteroside (2)	
	*δ*_C_	*δ*_H_, mult. (*J* in Hz)	*δ*_C_	*δ*_H_, mult. (*J* in Hz)
2	164.0		163.6	
3	121.6	5.98, d (9.6)	122.8	6.09, d (9.8)
4	146.8	7.01, dd (5.7, 9.6)	144.7	7.13, dd (9.8, 5.1)
5	61.5	3.93, m	67.1	4.36, dd (3.4,5.1)
6	77.1	4.48, dq (2.8, 6.6)	76.5	4.66, dq (3.4, 6.6)
7	16.1	1.29, d (6.6)	16.3	1.34, d (6.6)
1′			101.5	4.3, d (7.8)
2′			73.8	2.94–3.04, m
3′			77.3	3.12, m
4′			70.6	2.94–3.04, m
5′			77.6	3.12, m
6′			61.8	3.43, m

**Table 2:** Quantification of compounds **1** and **2** in *A. helferiana* rhizome using calibration curve.

Analytes	Regression Equation	Correlation Coefficient	Content in Dried Rhizome (mg/g of Dried Rhizome)	Content in Dried Rhizome (%)
Compound **1**	y = 4085.5x + 86.49	0.994	15.40	1.54
Compound **2**	y = 2232.7x + 77.64	0.994	32.05	3.20
